# Investigation of Embedded Si/C System Exposed to a Hybrid Reaction of Centrifugal-Assisted Thermite Method

**DOI:** 10.1371/journal.pone.0144632

**Published:** 2015-12-07

**Authors:** Reza Mahmoodian, Rosiyah Yahya, Ali Dabbagh, Mohd Hamdi, Mohsen A. Hassan

**Affiliations:** 1 Centre of Advanced Manufacturing and Materials Processing (AMMP), Department of Mechanical Engineering, Faculty of Engineering, University of Malaya, Kuala Lumpur, Malaysia; 2 Department of Chemistry, Faculty of Science, University of Malaya, Kuala Lumpur, Malaysia; 3 Department of Research and Development, Azarin Kar Ind. Co., Industrial Zone 1, Kerman, Iran; 4 Department of Mechanical Engineering, Assiut University, Assiut, Egypt; VIT University, INDIA

## Abstract

A novel method is proposed to study the behavior and phase formation of a Si+C compacted pellet under centrifugal acceleration in a hybrid reaction. Si+C as elemental mixture in the form of a pellet is embedded in a centrifugal tube. The pellet assembly and tube are exposed to the sudden thermal energy of a thermite reaction resulted in a hybrid reaction. The hybrid reaction of thermite and Si+C produced unique phases. X-ray diffraction pattern (XRD) as well as microstructural and elemental analyses are then investigated. XRD pattern showed formation of materials with possible electronic and magnetic properties. The cooling rate and the molten particle viscosity mathematical model of the process are meant to assist in understanding the physical and chemical phenomena took place during and after reaction. The results analysis revealed that up to 85% of materials converted into secondary products as ceramics-matrix composite.

## Introduction

The Self-propagating high-temperature synthesis (SHS) technique leads to the in situ production of composites from initial reactive substances through an exothermic chemical reaction [1]. The heat released by the reaction helps ignite and sustain a propagating combustion front through the reactants, thus creating the anticipated product [2]. SHS is distinguished by high temperatures, rendering it an alternative, more economical method for the ceramic industries compared to conventional ceramic processing. SHS is a feasible technique for manufacturing advanced ceramics, catalysts, and nanomaterials [2]. Combustion synthesis is a versatile means of synthesizing a variety of technologically useful solid materials, such as binary and ternary metal borides [3], carbides [4, 5], silicide, chalcogenides, and nitrides [6], hydrides [7], alloys [8], composites [9], cemented carbides [10], or composite materials [11] in a single processing step in contrast to conventional ceramic processing, which is among the main advantages of SHS processing [12].

Combustion synthesis reaction is known by the adiabatic combustion temperature T_ad_. The adiabatic combustion can be deliberated by assuming that the changes in enthalpy of the reaction heats up the products and no energy is lost by heating convection or radiation to the surrounding atmosphere. Thus, T_ad_ is an amount of the reaction’s exothermicity and shapes the upper frontier for any combustion system. Empirically, if T_ad_ < 1200°C, combustion does not occur, and if T_ad_ > 2200°C, self-propagating combustion happens. In the range of 1200< T_ad_ < 2200°C, a combustion wave cannot propagate however it can be made to do so by a exceptional method, such as pre-heating the reactants. The reaction (1) is self-sustaining unlike the reaction (2) where T_ad_ = 1527, T_ig_ = 1300°C [12].

$$Fe_{2}O_{3}+2Al\to Al_{2}O_{3}+2Fe+836\;kJ\;mol^{-1}$$ (1)

$$Si+C\to SiC+75\;kJ\;mol^{-1}$$ (2)

Some controls are applied to the highly exothermic combustion synthesis process including reactant particle shape and size, powder mixing and compaction, impurities, reaction stoichiometry, volatiles and diluents, reaction environment, ignition mode and technique, heating speed, and the effect of these parameters on heat generation, exothermicity, and SHS reaction mechanism [13–15].

Crystalline cubic silicon carbide (3C-SiC) is reportedly a wide-energy gap material with a number of excellent properties, among which are stability at high temperatures, high mechanical and chemical stability, and high irradiation resistance [16, 17]. Its equivalents, nanocrystalline SiC (β-SiC) and amorphous SiC (α-SiC) have similarly attracted substantial research attention. This is owing to their superior physical, chemical and electrical properties over nanocrystalline and amorphous Si and because they are potential materials for producing high-efficiency solar cells, light-emitting diodes, and they have been widely applied as thermal coating layers and diffusion barriers against both metal and dopant diffusion [18].

SiC has gained the attention of industries on account of its advantages including extreme hardness [19], high abrasive capability, high Young’s modulus, high temperature resistance up to 1500°C, and high resistance to abrasion [20]; though its high residual porosity imposes a limitation as far as mechanical strength is concerned [21]. Several processing methods have been proposed for SiC synthesis in open literature as found in a number of papers [22–28]. In this work, phase formation and separation are investigated by employing the thermal energy released in the form of the hybrid reactions of thermite and silicon carbide under centrifugal acceleration. To the best of the authors’ knowledge, no reports on silicon carbide system processing using the above-mentioned method exist. The proposed setup is significant for supplementary single-stage applications of locally reinforced ceramic-coated pipes and related fields.

## Materials and Methods

### Experimental setup

In this technique, the scheme is to investigate the formation of SiC during a centrifugal thermite process. The starting thermite materials, namely Al (< 75 μm, 99% purity, Sigma Aldrich) and Fe_2_O_3_ (< 5 μm, 97% purity, Sigma Aldrich) powders were dried for 8 hours at 110°C in a drier and mixed for 4 hours at 30-minute intervals according to Eq (1). Silicon (Sigma Aldrich, -325 mesh, 99% purity) and carbon (Sigma Aldrich, -1000 mesh, 99.9% purity) powders were prepared agreeing to the stoichiometry Eq (2). The Si+C pellet with the green density of 1.53 g/cm^3^ was embedded in a compacted graphite mold within a steel tube. The pellet was situated in a region near the tube’s head in order to be subjected to a higher thermal gradient [29]. The tube was mounted in a centrifugal machine whose processing mechanism is explained in recent publications [30].

**Fig 1:**
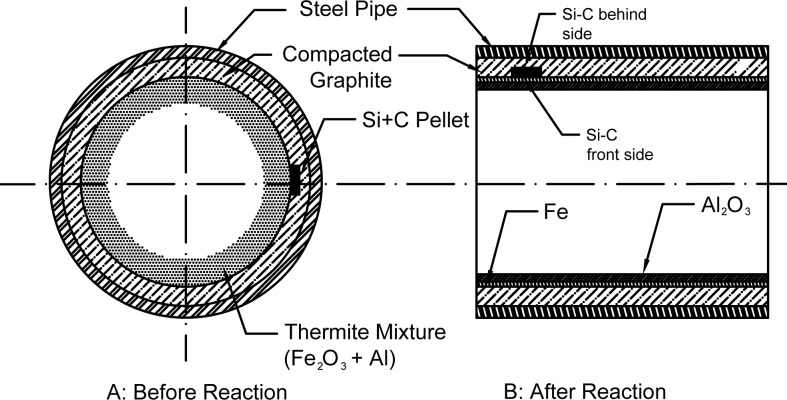
The schematic illustrating: Si+C embedded centrifugal thermite and a compacted graphite crucible assembly charged with the loaded green mixture A: before reaction (left), B: after reaction (right) while subjected to centrifugal force, the front and behind side of Si-C pellet is identified after reaction.

Preliminary experiments showed that the centrifugal thermite temperature increased up to 2800°C [31]. The temperature was recorded a high performance infrared thermometer (pyrometer), Raytek MM1MHSF3L. Therefore, this experiment was set up according to the setting revealed in Fig 1. The green thermite mixture of reaction (1) was fed into the rotating tube at 5 *g* acceleration. Then, the mixture was ignited and finally, the product was removed from the chamber for further characterization. The infrared pyrometer recorded the system’s real-time temperature during the process.

The microstructure and the characteristic peaks of the samples were determined by field emission scanning electron microscopy (Gemini FESEM; Carl Zeiss- Auriga 60 microscope, Jena, Germany) and, X-ray powder diffractometer (XRD, PANalytical's Empyrean) with a monochromated CuKα radiation (λ  = 1.54056 Å), which was operated at 45 kV and 40 mA with a step size of 0.026 deg and a scanning rate of 0.1 deg s^−1^ in the 2θ range of 10 to 90 deg. The Rietveld method [32] was used to calculate the phase contents (quantitative phase analysis) in the specimen. In the Rietveld method, crystal structure and peak profile parameters are refined in several stages. Inorganic Crystal Structure Database (ICSD) and Crystallography Open Database (COD_Oct2014) were used as the reference library to match peaks. The HighScore Plus 3.0d was employed to do the processing of the Rietveld refinement stage calculations.

### Mathematical Modeling of the Hybrid SHS Mechanism

The effect of thermite energy (heat) on a Si+C pellet under centrifugal acceleration is elaborated in this section. The current method was employed to process a Si+C pellet at high centrifugal force. The thermite mixture liberated a vast amount of energy in the form of heat upon ignition. The reaction’s chemical formula and released thermal energy is given in Eq (1). The proposed mathematical model will demonstrate molten particle segregation of Fe and Si-C pellet. It is intended to employ the generated heat to process a Si+C pellet and convert it into a secondary product, desirably a silicon carbide (SiC).

#### Model description

**Fig 2:**
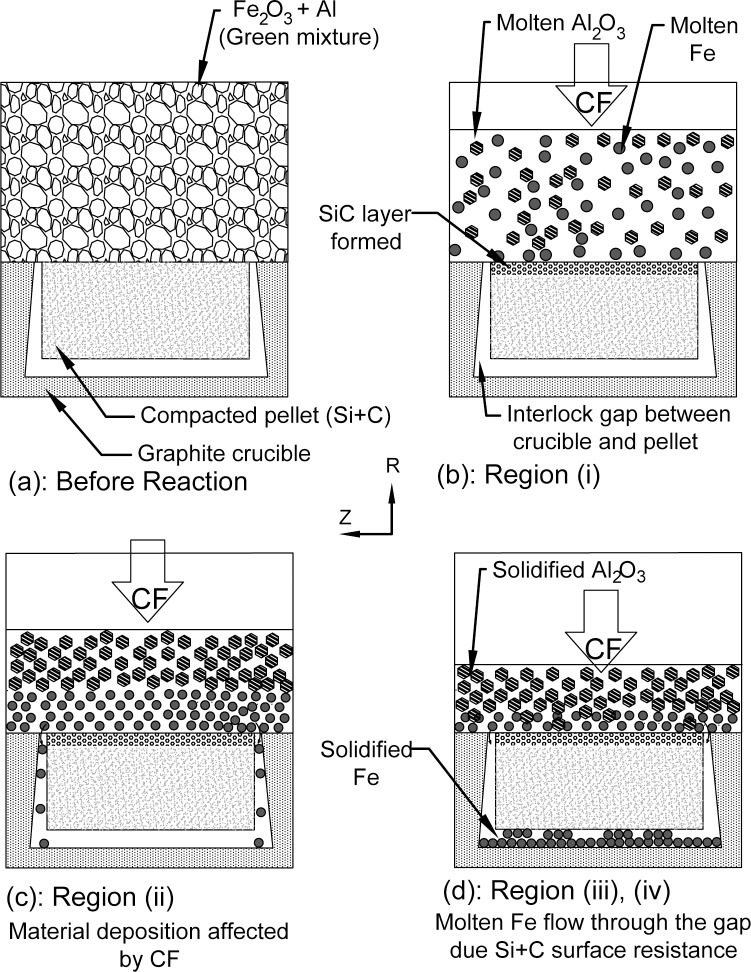
The effect of Centrifugal Force (CF) on the material particles during the centrifugal SHS coating process; (a) before thermite reaction ignition; (b) at the time of initial reaction; (c) molten material about to be deposited onto the pellet; and (d) solidified particles after deposition onto the surface.

**Fig 3:**
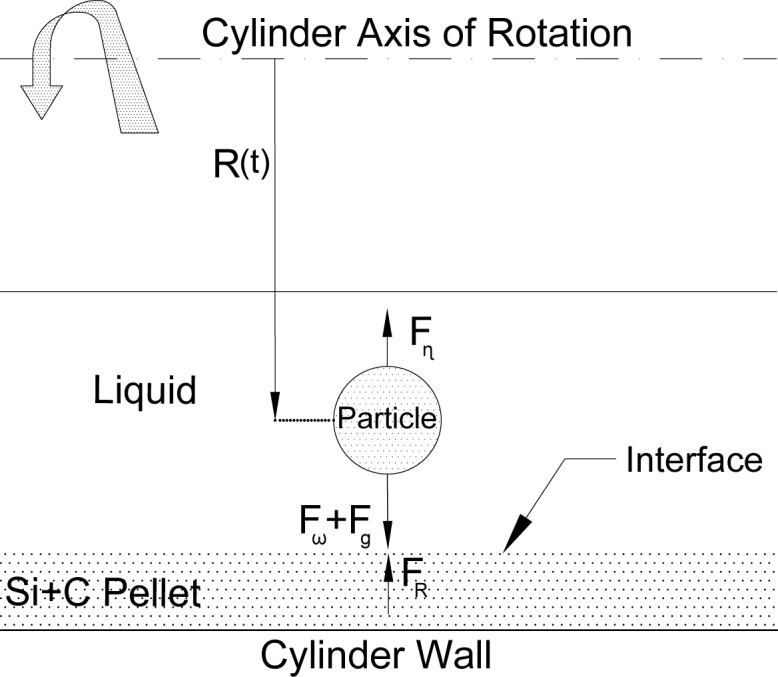
Schematic representation of forces acting on a moving particle during the hybrid centrifugal SHS process.

**Fig 4:**
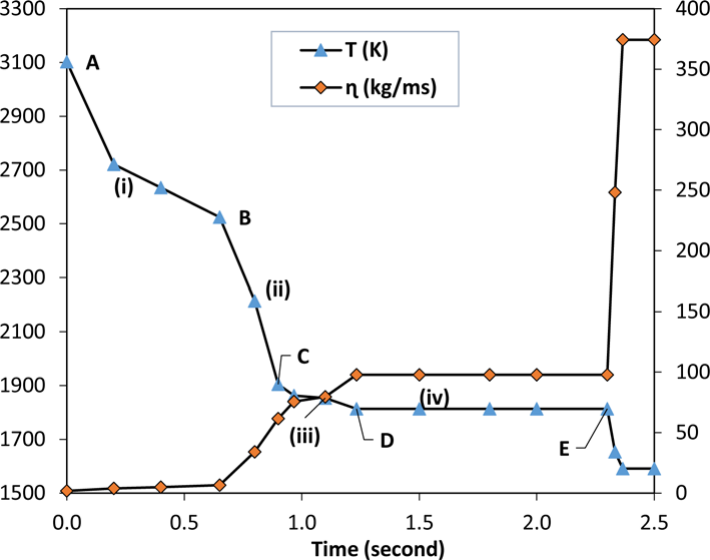
The pyrometer temperature reading and calculated values of viscosity versus time during the hybrid SHS process; d = 1.00E-04 m, n = 58 RPS, ρ_o_ = 7800 kgm^-3^, ρ_m_ = 2700 kgm^-3^, R = 2.50E-02 m. Region (i) shows a sudden temperature decline immediately after the reaction started (point A to B), region (ii) temperature drops to the melting point of Fe the point B to C. At region (iii), (iv) the crucible temperature stays steady for 1.5 s around Fe melting point, the solidification of the molten Fe starts at point E.

Diagram of the materials deposition are illustrated in Figs 2 and 3. Fig 2(A) shows compacted Si+C powder and some thermite mixture prior to the reaction occurrence. Fig 2(B) corresponds to region (i) of Fig 4, describing the reaction during exposure to centrifugal force (CF). The green mixture has reacted and produced molten Al_2_O_3_ and Fe while the generated heat led to the initiation of the SiC reaction.

Fig 2(C) shows the particles’ motion during the solidification process of Al_2_O_3_ that occurs in region (ii) of Fig 2. At this stage, the molten Al_2_O_3_ has lost its heat and solidified, whereas the molten Fe will infiltrate into the SiC porous site due to the high CF. The CF for a given mass of particle (m_p_)_,_ revolutions per second (n) and the particle’s position (R) is calculated as *CF* = 39.4*m*_*p*_ *Rn* ^2^ if the gravity force is neglected [33].

Fig 3 presents the schematic of four forces acting on a particle during the centrifugal SHS coating process. Since the rotation axis is horizontal, the centrifugal and gravity forces work in the same direction and perpendicular to the cylinder axis of rotation. Viscosity (F_ɳ_) and repulsive (F_R_) forces work against the particle’s motion towards the cylinder wall and on the same line of action as the centrifugal force. Therefore, the summation of forces acting on a particle at position R(t) from the horizontal axis and near the solid-liquid interface region is given as:

$$F_{\omega }+F_{g}-F_{ɳ}-F_{R}=F_{nett}$$ (3)

Where Fω, F_g_, F_ɳ_, F_R_, F_nett_ represent centrifugal force, gravity, viscosity and repulsive forces, respectively. F_R_ in Eq 3 cannot be neglected according to field emission scanning electron microscopy (FESEM) and energy dispersive x-ray analysis (EDS) observations. However, it is only significant when the particle is close to the solid-liquid interface [34].

#### Calculations of particles viscosity

Eq 4 which is derived from the Arrhenius equation calculated the initial viscosity of molten steel, A = 0.0065 kg/ m^.^ s [35], the gas constant is R_g_ = 8.31441 J/ K.mol [34], and the activation energy Q = 145 kJ/mol [36]. The measured temperature versus time in Fig 4 was used to calculate the current viscosity ɳ(Tc) of molten metal prior to calculating the deposition velocity. This procedure was repeated to solve Eq 4 for a time range of 0 to 2.5 s and a temperature range of 3100 to 1550°K.

$$\eta \left(T_{c}\right)=A\;\exp\;\left(\frac{Q}{R_{g}.T_{c}}\right)$$ (4)

The calculated values of the system’s viscosity versus time and real-time temperature readings are plotted in Fig 4. A rapid heating occurred at 0 seconds, followed by a fast cooling progression from 0 to 2.5 s in a single step of the reaction to produce Al_2_O_3_-Fe composite followed by SiC formation. The general trend of the viscosity curve increases with time as the relative temperature decreases.

In the time range of 0 to 0.2 s, which corresponds to region (i) in Fig 4, the particles’ liquid viscosity sharply increases. In region (ii) the particle viscosity ɳ (Tc) continues to show a significant increase with time up to 1.0 s, since the Al_2_O_3_ particles are solidified more rapidly. Though, in region (iii), from 1.0 to 2.3 s ɳ (Tc) does not significantly change. This phenomenon can be attributed to the constant infiltration rate of Fe particles toward the SiC product. In region (iv), after 2.3 s, due to the temperature dropped to 1800°K, the relative viscosity increased from 97.3 to 376 kg m^-1^ s^-1^ thus stopping any further particle movement as a liquid. However, solid-state diffusion may still occur and steel phase changes happen.

## Results and Discussion

**Fig 5:**
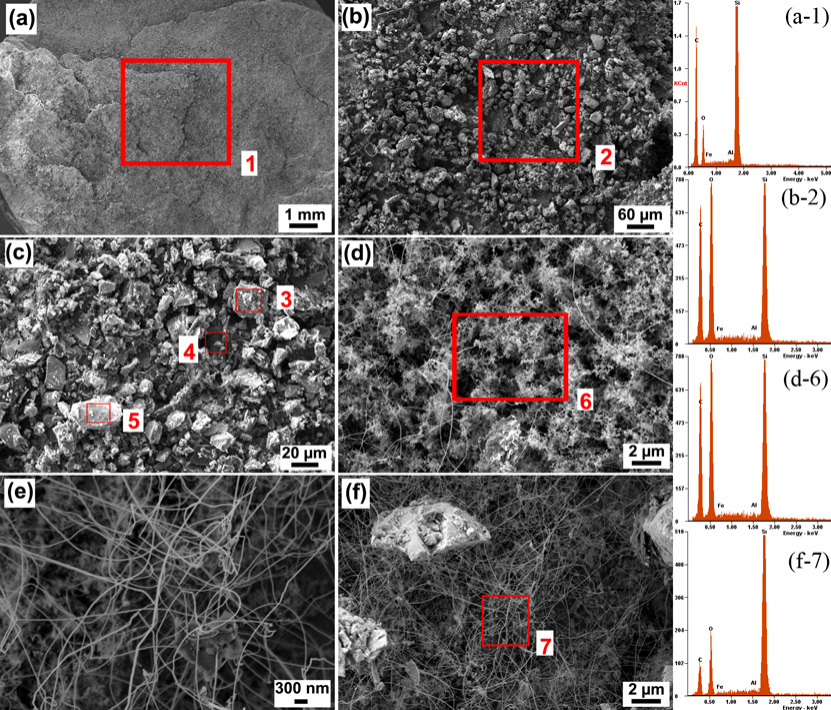
FESEM micrographs and EDS elemental analyses of a Si-C pellet removed from the tube of a centrifugal thermite-assisted reaction: (a) overall topography, (b), (c), and (d) microstructure of typical points; (e) and (f) high magnification of region (d).

**Fig 6:**
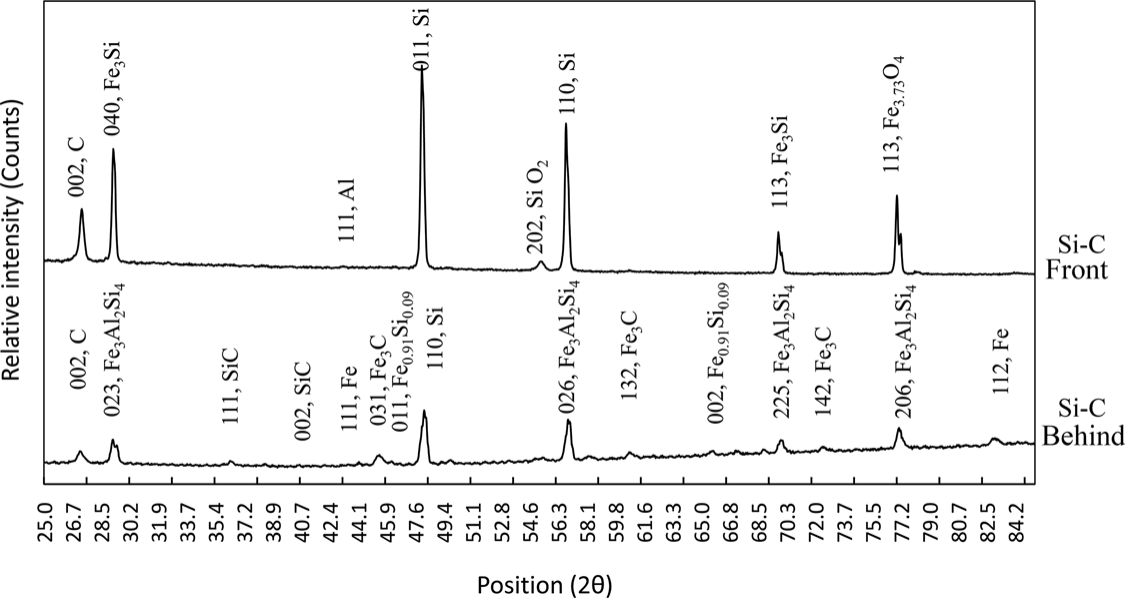
XRD patterns of the Silicon-Carbon pellet after being exposed to centrifugal SHS.

The formation of silicon carbide iron composite was attempted using the thermite reaction energy under centrifugal acceleration. Visual observation of the as-sintered specimen that was removed from the crucible revealed that the Si+C pellet bonding following the reaction was too loose and could be crushed easily with the least amount of force against a paper. The microstructure field emission scanning electron microscopy (FESEM) and corresponding elemental analysis, Energy Dispersive X-ray analysis (EDS), and X-ray diffraction patterns (XRD) of the specimen after undergoing the thermite reaction are featured in Fig 5, Table 1, and Fig 6, respectively.

**Table 1:** EDS elemental analysis of different points on the Si+C pellet.

Part figure	Spotted area	Si	C	O	Al	Fe
(a)	1	46.05%	47.07%	6.10%	0.47%	0.31%
(b)	2	51.34%	45.01%	3.02%	0.41%	0.22%
(c)	3	88.15%	11.05%	0.80%	-	-
(c)	4	56.19%	33.03%	9.27%	0.80%	0.71%
(c)	5	83.82%	8.83%	6.22%	0.67%	0.46%
(d)	6	52.31%	24.36%	22.16%	0.75%	0.42%
(f)	7	80.58%	9.97%	8.12%	0.90%	0.43%

The overall morphology of the specimen is depicted in Fig 5(A) at low magnification. From a macroscopic point of view, a non-smooth structure with many defects and cracks is evident. These imperfections are consequent to repositioning the specimen from the crucible owing to its very loose structure. Fig 5(B) and 5(C) illustrate closer views of the presented structure, revealing the morphology of micro crystals. The elemental analysis (EDS) of various regions shows that the compound has a non-uniform distribution with region (3) presenting no Al despite the other two regions, (4) and (5), having less than 1% Al content. Fig 5(D), 5(E) and 5(F) indicate that the specimen includes mainly unstructured content with networks of nanowires and whiskers. The nanowires’ net shape and orientations signify that centrifugal force has assisted the wire formation in a directional path from the bottom toward the top of the specimen (Fig 1). From the elemental analysis (Table 1), the material seems mostly to be an oxide phase. It is probable that silicon oxide nanowires were mostly formed in the interfacial location.

The X-ray diffraction patterns (XRD) of the both sides of the Si-C pellet are shown in Fig 6. The face exposed to thermite heat is called the “front side” and the face that was not directly exposed to thermite heat, but was affected by molten Fe flowing from the side of the pellet between the pellet and crucible’s engraved area is known as the “behind side”. The arrangement of the sides of the pellet is illustrated in Fig 1. The corresponding phase quantification and powder diffraction file (PDF) code of both sides are presented in Table 2. The related elemental (EDS) and XRD analyses revealed that oxide formation potentially occurred mainly in the form of silicon dioxide at the front side which was faced to direct thermite reaction and high temperature fume atmosphere.

**Table 2:** Phase quantification of the as-sintered Si-C specimen at the front (faced to thermite reaction) and back (exposed to molten Fe) sides.

XRD scan locations	Phase	PDF code	Rietveld Quantification
Front side	C	COD 96-110-0004	19.90%
	Si O_2_	ICSD 98-017-2290	38.70%
	Fe_3_Si	ICSD 98-041-2838	3.10%
	Fe_3.73_O_4_	COD 96-101-1169	1.90%
	Si	COD 96-901-2920	24.60%
	Al	COD 96-431-3211	11.00%
	Fe	COD 96-900-0665	0.80%
Back side	Fe_3_C	COD 96-101-0937	26.08%
	C	COD 96-110-0004	6.10%
	Si	COD 96-901-2920	8.60%
	Si C	COD 96-101-0996	8.30%
	Fe_0.91_Si_0.09_	COD 96-900-6623	7.22%
	Fe_3_Al_2_Si_4_	COD 96-200-5763	23.80%
	Fe	COD 96-901-3464	19.90%

As per Eq (2), the reaction between Si and C elemental powders is less exothermic than the thermite reaction (Eq (1)). Nevertheless, the adiabatic temperature was insufficient to propagate along the front side of the sample, unless the thermal environment was satisfied [37]. Despite a similar experimental setup to the one employed in TiC processing [31, 38], the Si-C pellet did not actively partake in the hybrid reaction following the thermite reaction.

This can be seen from the Fig 5, as here is no significant phase such as SiC, AlSi, etc at the front side were detected in the XRD pattern. The profile shape of the XRD pattern reveals that the specimen may have amorphous content. Amorphous, or poorly crystalline materials do not contribute to diffraction peaks, and thus it is not possible to determine the quantification with the Rietveld refinement [39–41]. Therefore, Table 2 illustrates only the polycrystalline material portion as established by Rietveld quantification. The phase formation at the behind side of the specimen compared more evident with the front side. The most significant phase change that occurred in the specimen was SiO_2_ and Fe_3_C, SiC, Fe_3_Al_2_Si_4_ for front and behind-side, respectively. Again, this phenomenon is a result of the rapid heat dissipation, which disallows crystallization for the front-side and also molten Fe trap at the behind-side of the specimen.

Moreover, the XRD patterns shows a few peaks containing with Fe constituent (alloy) at 2theta = 29.2, 69.8, 77.0 degree on Si-C front and at 50, 61, 70, 72.4, 77, 83.1 on Si-C behind. There is no significant pure Fe phase observed at Si-C front while some are found in SiC-behind pattern. The XRD analysis shows 55% and 85% phase formation has happened in case of Si-C front and Si-C behind, respectively.

The presence of minute amounts of Al suggests that it is not possible to obtain pure phase formation. The significance of tracing Al throughout the specimen is to recognize the phase formations and particle segregations in different zones by the help of EDS. As demonstrated in previous research work, the segregation of metal impurities is the principle mechanism for Al happens due to the higher solubility in the Al-Si layer [42].

Though iron is reported to be highly soluble in liquid aluminum and its alloys, it has very slight solubility in the solid state (max. 0.05 wt%, 0.025 atom %) and so it tends to combine with other elements to form intermetallic phase particles of various types. In the absence of Si, the dominant phases formed are Fe_3_C, but in the presence of Si, as in the most widespread foundry alloys, Orthorhombic Fe_3_Al_2_Si_4_, cubic Fe_0.91_Si_0.09_ and Fe_3_Si phases are dominant [43]. However, in this experiment aluminum is consumed in the iron reduction from Fe_2_O_3_. Consequently, the majority of the starting elemental Al is converted into alumina at the tube. In accordance with XRD analysis, there were small amounts of Al traced throughout the specimen front-side as elemental material along with iron oxide, which are assumed to be unreacted starting materials. Moreover, at the specimen behind side, some of the Al diffused into the Si+C pellet and formed an intermetallic compound of Fe_3_Al_2_Si_4_. The Al containing an intermetallic compound is 23.8% according to XRD results. We would like to emphasis that the Rietveld phase quantification is not absolute due to the existing faint peaks.

It is evident that the Si-C combustion synthesis reaction requirements were not completely satisfied in order to instigate the reaction and propagate along the specimen. This behavior is in agreement with that reported by Schubert and Hüsing [12]. As explained earlier, SHS reactions are characterized by adiabatic combustion temperature T_ad_ that can be calculated assuming that the reaction enthalpy heats up the products and no energy is lost by heating the surrounding environment. For self-propagating combustion to occur, T_ad_ must be within the 1200–2200°C range.

The XRD result also confirms that the heavier material composed of Fe and its intermetallic compound was caught on the behind side of the pellet (Fig 2) due to the direct effect of centrifugal acceleration. Whereas, in accordance with mathematical calculations (Eq 4), the corresponding results, the viscosity and temperature plot (Fig 4), the iron phase had been in a liquid state longer than alumina. Subsequently, this phenomenon has helped the molten iron particles to diffuse and flow around the specimen as shown in Figs 2 and 3.

In this work, it was not feasible to apply a pre-heating stage before the reaction. Since this technique is an amalgamation of centrifugal thermite and SiC SHS processes, which can lead to a premature reaction and cause catastrophic incidents regarding safety during rotation at high velocity. Therefore, synthesizing SiC from the thermite reaction heat under centrifugal acceleration using this technique does not produce high amount of pure SiC, but a composite structure of Si-Fe/SiC/Fe_3_C/Fe_3_Al_2_Si_4_ is achieved. According to Schmalzried and Schwetz [44] 3C-SiC silicon carbide is formed between 1400 and about 1600°C, and 15R-SiC above 2200°C.

In accordance with the infrared pyrometer reading, the temperature attained was over 2200°C. Therefore, the most significant phase changes were found at the behind side of the specimen. As the melting point of Fe is 1538°C and according to the temperature reading information (Fig 4), the system temperature was steady at this level for around 2 seconds. Consequently, 3C-SiC is formed which is confirmed by XRD as well. Therefore, high Fe rich phases are formed on the behind side of the specimen. However, there are always solid diffusion of particles happens during solicitation and below the melting points of the materials and this phenomena usually results in intermetallic compounds formation [45]. According to Odkhuu and Soon Cheol [46], [47], the current multiphase product may have electronic and magnetic properties.

Nevertheless, the reaction occurred in the atmosphere of an argon-purged chamber. Consistent with XRD analysis, oxygen gas remained inside the chamber. Additional oxygen could enter the chamber, as the pipe head (or chamber cap) was kept open for the process devices to make contact. Thus, phases of silicon dioxide are detected in the XRD pattern. Technically, it was not viable to evaluate the mechanical properties of the processed Si-C owing to the highly porous structure. The results of the experiments shows formation of a composed system of products, containing SiC.

## Conclusions

A composite of Si-Fe/SiC/Al_2_O_3_ was achieved due to semi self-sustainable reaction. Around between 55–85% of the starting materials were converted into secondary products. The mathematical model could help to justify the effect of molten iron and particle segregation during the process which affected more of phase formation at behind side of the pellet. Varying material structures were detected in XRD which is in agreement with the mathematical modeling.
